# Plumage color degradation indicates reproductive effort: an experiment

**DOI:** 10.1038/s41598-023-45348-0

**Published:** 2023-10-31

**Authors:** Gergely Hegyi, Miklós Laczi, Gyula Szabó, Fanni Sarkadi, János Török

**Affiliations:** 1 Behavioral Ecology Group, Department of Systematic Zoology and Ecology, ELTE Eötvös Loránd University, 1117 Pázmány Péter sétány 1/C, Budapest, Hungary; 2 HUN-REN-ELTE-MTM Integrative Ecology Research Group, 1117 Pázmány Péter sétány 1/C, Budapest, Hungary; 3 Barn Owl Foundation, Temesvári út 8, 8744 Orosztony, Hungary

**Keywords:** Behavioural ecology, Sexual selection

## Abstract

Plumage color has traditionally been regarded as a static ornamental trait, but evidence is accumulating for significant color changes without molt that typically reduce the conspicuousness of ornamentation. In some species, the social partner seems to increase its reproductive investment if the color trait is experimentally enhanced, suggesting that color change could act as a signal. However, the information content of this signal is so far unclear. For example, birds in poor condition or making greater effort may deteriorate more severely. We used brood size manipulations to alter the reproductive effort of male and female collared flycatchers *Ficedula albicollis*. Both sexes showed less severe decline in some reflectance attribute of their white breast when their brood was experimentally reduced. In each sex, greater deterioration of the reflectance trait affected by the manipulation was accompanied by increased feeding rate by the partner. These feeding patterns do not prove, but are consistent with, a compensatory response by the partner to induced degradation. The manipulation effects on color change we detected confirm for the first time that plumage color deterioration can indicate current reproductive effort, thereby providing a potential fitness advantage to social partners that react to such deterioration.

## Introduction

A central question concerning sexual ornaments is the source of their reliability that provides sufficient benefits for the chooser to counterbalance the costs of considering the signal^1^. Plumage coloration is a well known target of sexual selection, and it is typically categorized based on the proximate background of individual differences. Pigment-based color gets its information content from pigment quality or quantity^2,3^. Chromatic structural coloration (typically UV-blue or iridescent) involves the generation or self-assembly of ordered nanostructures and it may therefore gain its information content from the precision of such structures^4^. The most contentious type of coloration is white plumage patches. These are widespread in many taxa^5–7^, and have been repeatedly shown to indicate aspects of individual quality such as condition, parasite load and reproductive output^8–10^. These patches contain neither pigment nor ordered nanostructure^11^, so they must gain their reliability from the quantity or quality of the reflective surfaces^12^.

However, information content and reliability are more complicated than this because plumage color does not seem to be a static trait. Even though typically one or a maximum of two molts are performed each year^13^, coloration has been shown to change without feather replacement. Its conspicuousness may increase^14,15^, but it typically decreases^16,17^. Plumage color change between molts may confound analyses of signal information content or function^18^ or delimit the most informative period of the trait^15^. Moreover, partners seem to respond to color changes. Within-season manipulations of the coloration of a breeding bird seem to induce changes in parental investment by the partner^19,20^. Color change may constitute a signal of condition or quality for the social mate or even the neighbor. However, this point has not been confirmed yet, as no study to our knowledge has experimentally demonstrated the information content of within-season plumage color shifts, that is, the reason why the observed changes in partner investment would be adaptive.

In white plumage areas, the lack of feather-strengthening melanin^21^ disproportionately exposes feather structure to mechanical abrasion and therefore potentially to color degradation^22,23^. Therefore, white area reflectance is an ideal candidate for dynamic signaling. Here we report on experimental manipulations of brood size where we assessed the change of reproductive effort and the immediate degradation of a sexually selected white ornamentation system in response to the manipulation. We conducted the study in collared flycatchers (*Ficedula albicollis*). In the study population, the reflectance of white plumage areas is sexually selected as part of a plumage-level integrated ornamentation system, where mating patterns follow the overall brightness and relative ultraviolet reflectance of the plumage in both sexes^24^. Differential degradation of color across the plumage has been suggested to occur, frequenting the areas more exposed to damage, particularly the breast^18^. This also influences the integration and functioning of the ornamentation system^25^. Here we applied experimental manipulations of brood size^26,27^ to clarify the causal effect of reproductive effort, and studied the degradation of color during nestling rearing across three major white areas of the plumage: wing patch and breast in both sexes, and forehead patch in males. Previous studies suggested area-dependent degradation that presumably reflects different structural background and differential exposure to damage in different plumage areas^18^, so we conducted this study at the level of individual white areas. We predicted that experimentally increased reproductive effort would produce increased degradation and reduced reproductive effort would lead to reduced degradation, especially in the breast which is the most vulnerable to damage. We also observed own and partner feeding patterns in relation to ornament degradation in the late nestling stage to see whether these patterns are consistent with the assumption that social partners adjust their apparent feeding activity to the effort-related degradation of their mate’s reflectance. This latter analysis is tentative but necessary. If we find no relationship between the degradation of the trait affected by the manipulation and partner feeding in this scenario where both trait change and partner feeding have increased variance due to the experiment, then this falsifies the assumption that the trait is used as a signal by the other sex. In such a case, any conclusion concerning “signal information content” would have to be treated with caution. If, on the other hand, the analysis detects a significant relationship between trait change and partner feeding, then we cannot exclude the possibility that trait change is a signal. In this case, it is valid to talk about the information content of this putative signal trait, but further experiments, involving direct color manipulation, will be necessary to confirm that the trait is indeed used as a signal.

## Results

### Manipulation, reproductive effort and output

The feeding rate of parents reacted to manipulation in both sexes, but in a year-dependent manner (Table 1). In 2021, the enlarged group showed greater feeding rate from both others (Scheffé tests, p < 0.001) while reduced broods were fed less often than controls but the difference was not statistically significant (p = 0.120). In 2022, only the reduced group (lower feeding rate) differed from controls (p = 0.021) and tended to differ from enlarged ones (p = 0.096), while control and enlarged broods were very similar (p = 0.880). The total mass of all nestlings before fledging (nestling biomass production) also depended on manipulation in a year-dependent manner (year $\times$ manipulation F_2,40_ = 3.71, p = 0.033). In 2021, all manipulation categories differed in the expected direction (reduced > control > enlarged; manipulation F_2.18_ = 35.12, p < 0.001; all Scheffé tests p < 0.013). In 2022 (F_2,22_ = 7.52, p = 0.003), reduced broods produced less biomass (reduced vs. control p = 0.007, reduced vs. enlarged p = 0.020), but control and enlarged broods were very similar (p = 0.999). The initial body mass of parents depended on sex but not any other predictor (Table 1). The body mass changes of both sexes were negative overall during nestling rearing, but they were far smaller in males than in females (manipulation groups pooled, paired t tests, Table 2). When corrected for sex-standardized initial body mass, body mass change showed significant effects of sex, year and manipulation, but no interaction whatsoever between these (Table 1). The effect of manipulation was caused by differences between the reduced and the other groups (reduced > control p = 0.015, reduced > enlarged p < 0.001), with little difference between enlarged and control groups (p = 0.288). In sum, patterns in nestling biomass production, feeding rate and the body mass changes of the parents all indicate that brood size reduction successfully altered reproductive effort but we cannot state this with similar certainty for brood size enlargement.

**Table 1:** Original body mass, body mass change and feeding rate of parents in relation to sex, year, manipulation and their interactions. Body mass change is corrected for original body mass by including it as a covariate. *p < 0.05; **p < 0.01; ***p < 0.001; NA, not applicable.

	Original mass		Mass change		Feeding per hour	
	F	df	F	df	F	df
Sex	99.35***	1, 83	126.92***	1, 78	0.94	1, 91
Year	0.29	1, 82	9.79**	1, 78	4.11*	1, 92
Manipulation	2.83	2, 81	6.35**	2, 78	16.08***	1, 92
Sex $\times$ Year	1.56	1, 81	0.11	1, 77	3.38	1, 90
Sex $\times$ Manipulation	1.46	2, 79	0.26	2, 76	0.94	1, 89
Year $\times$ Manipulation	0.35	2, 78	0.23	2, 76	5.40**	1, 92
Sex $\times$ Year $\times$ Manipulation	1.98	2, 78	0.33	1, 77	1.44	1, 89
Original mass	NA	NA	30.65***	1, 78	NA	NA

**Table 2:** Changes of raw body mass and color variables of parents from 2d (Mean 1, SD 1) to 10d (Mean 2, SD 2) of nestling age: paired t tests with the manipulation categories pooled. *, p < 0.05; **, p < 0.01; ***, p < 0.001; NA, not applicable; SD, standard deviation.

Sex	Area	Trait	t	df	2 days of age		10 days of age		
					Mean	SD	Mean	SD	Unit
Male	NA	Body mass	5.405***	36	12.95	0.54	12.51	0.55	Gram
Male	Forehead patch	Brightness	5.142***	37	46.19	6.23	40.76	6.20	Percent
Male	Forehead patch	UV chroma	5.522***	37	0.73	0.06	0.67	0.07	Unitless
Male	Wing patch	Brightness	2.778**	37	41.44	5.28	39.26	4.11	Percent
Male	Wing patch	UV chroma	4.468***	37	0.87	0.04	0.83	0.05	Unitless
Male	Breast	Brightness	1.331	37	44.19	6.14	42.67	4.49	Percent
Male	Breast	UV chroma	1.902	37	0.86	0.06	0.83	0.08	Unitless
Female	NA	Body mass	18.828***	46	14.17	0.58	12.77	0.57	Gram
Female	Wing patch	Brightness	0.654	47	31.06	4.69	30.59	4.43	Percent
Female	Wing patch	UV chroma	7.776***	47	0.86	0.05	0.80	0.05	Unitless
Female	Breast	Brightness	1.257	47	37.37	6.92	36.00	5.60	Percent
Female	Breast	UV chroma	2.562*	47	0.74	0.12	0.69	0.11	Unitless

### Color degradation and reproductive effort

During nestling rearing, plumage color decline was prevalent across the white areas (manipulation groups pooled, paired t tests, Table 2). Breast brightness did not systematically decline in either sex, while wing patch brightness declined in males but not in females. Breast and wing patch UV chroma declined in both males (breast only marginal) and females. Finally, the forehead patch of males systematically degraded in both brightness and UV chroma.

**Figure 1:**
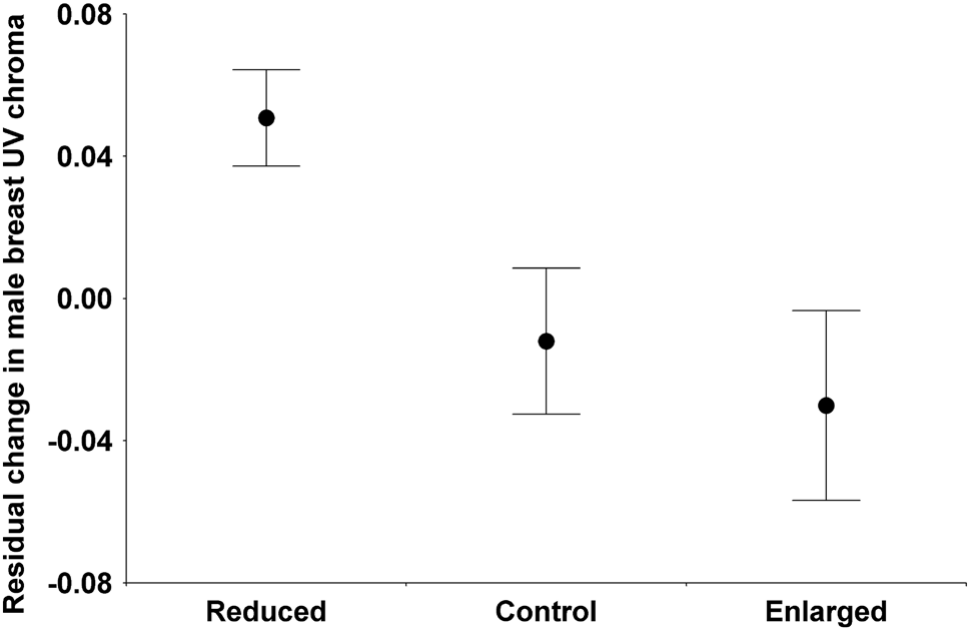
Change in the breast UV chroma of males in relation to brood size manipulation. Change is corrected for original value by calculating linear model residuals. The number of data is 11 for reduced, 14 for control and 13 for enlarged broods.

**Figure 2:**
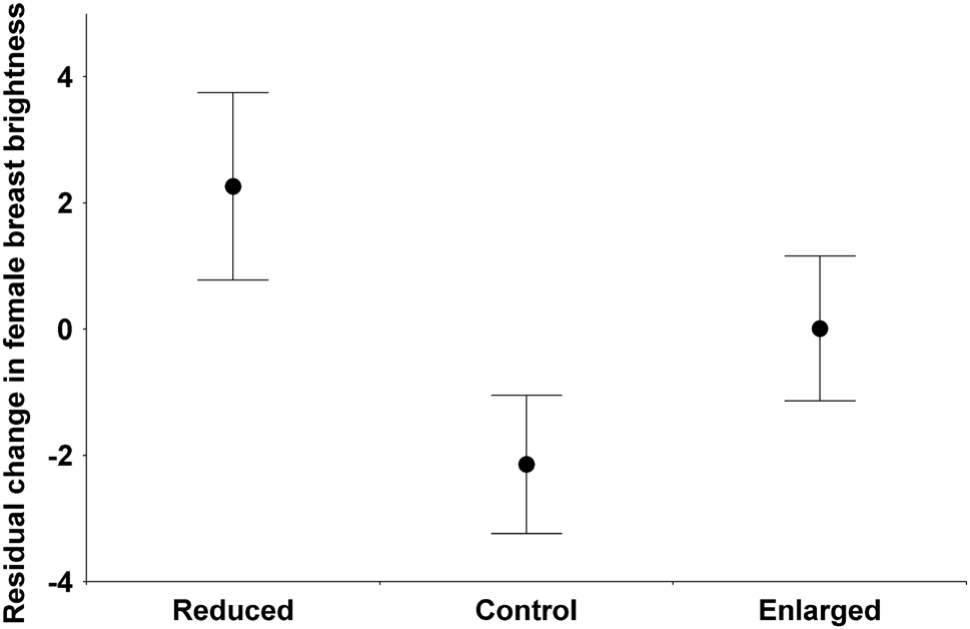
Change in the breast brightness of females in relation to brood size manipulation. Change is corrected for original value by calculating linear model residuals. The number of data is 17 for reduced, 18 for control and 13 for enlarged broods.

Before the manipulation, there was no difference between the manipulation categories in any of the white color traits of either sex irrespective of year (Table 3). Changes of reflectance after manipulation (corrected for original value, all details in Table 3) yielded manipulation effects for breast UV chroma in males (Fig. 1). The reduced group differed from the control (p = 0.039) and enlarged groups (p = 0.017) but control and enlarged groups did not differ (p = 0.619). There was also a manipulation effect on breast brightness in females (Fig. 2). The reduced group differed from controls (p = 0.003) but other comparisons were not significant because the enlarged group was intermediate (reduced vs. enlarged p = 0.285, control vs. enlarged p = 0.210). No significant manipulation effect was found for other white color traits, and the manipulation $\times$ year interaction was not significant for any trait. Finally, if we pool the control and the enlarged groups for the analysis because their reproductive effort has not been shown to differ (i.e. comparing reduced broods to all other broods), the same results are obtained, with male breast UV chroma and female breast brightness but no other trait significantly affected by the manipulation. This alternative analysis is shown in Supplementary Table S1.

**Table 3:** Original color variables and their changes in relation to year, manipulation and their interaction. Color change is corrected for original color by including it as a covariate. *, p < 0.05; **, p < 0.01; ***, p < 0.001.

			Original color before manipulation						Change in color after manipulation							
			Year		Manipulation		Year $\times$ manip		Year		Manipulation		Year $\times$ manip		Original color	
Sex	Area	Trait	F	df	F	df	F	df	F	df	F	df	F	df	F	df
Male	Forehead patch	Brightness	4.39*	1, 36	**0**.41	2, 34	0.23	2, 32	0.12	1, 35	1.58	2, 34	0.33	2, 31	13.95***	1, 36
Male	Forehead patch	UV chroma	1.14	1, 36	**0**.19	2, 35	2.30	2, 32	0.S2	1, 35	1.53	2, 34	0.42	2. 31	8.44**	1, 36
Male	Wing patch	Brightness	3.92	1, 36	1.44	2, 35	0.26	2, 32	0.29	1, 35	1.89	2, 34	2.55	2. 31	29.73***	1, 36
Male	Wing patch	UV chroma	3.14	1, 36	**0**.30	2, 35	0.48	2, 32	1.75	1, 35	2.77	2, 34	0.57	2. 31	9.20**	1, 36
Male	Breast	Brightness	4.09	1, 36	**0**.07	2, 35	0.70	2, 32	1.71	1, 35	0.3S	2, 34	0.15	2,31	54.87***	1, 36
Male	Breast	UV chroma	0.26	1, 36	**0**.44	2, 35	0.91	2, 32	0.27	1, 33	3.54*	2, 34	2.51	2, 31	10.93**	1, 34
Female	Whig patch	Brightness	0.92	1, 46	**0**.53	2, 45	0.49	2, 42	1.35	1, 45	**0**.12	2, 43	0.62	2,41	22.82***	1, 45
Female	Whig patch	UV chroma	0.00	1, 46	**0**.36	2, 45	0.91	2, 42	0.11	1, 45	**0**.79	2, 43	0.05	2,41	30.38***	1, 45
Female	Breast	Brightness	14.16***	1, 46	1.53	2, 44	0.35	2, 42	1.18	1, 43	3.28*	2, 44	0.10	2. 41	41.33***	1, 44
Female	Breast	UV chroma	0.33	1, 46	**0**.29	2, 45	0.10	2, 42	0.18	1, 45	2.11	2, 44	0.38	2.41	41.02***	1, 46

### Color degradation and feeding patterns

**Figure 3:**
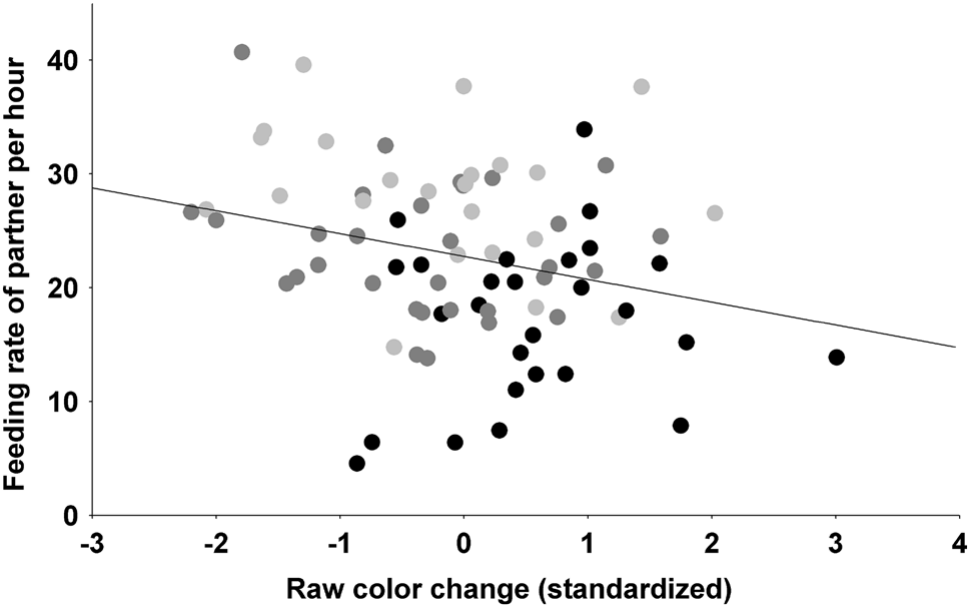
Relationship between raw color change of the manipulation-responsive trait of the given sex (standardized for sex) and the feeding rate of the partner at 10d of nestling age. Black, reduced broods; dark grey, control broods; light grey, enlarged broods.

Partner feeding rate was significantly negatively related to changes in the color trait that responded to the manipulation in the given sex (Fig. 3), but there was no effect of sex, the non-responsive trait of the signaler or the sex $\times$ trait change interactions (Table 4, first block). When we repeated the same analysis with change values corrected for pre-manipulation value, no parameter was significant (Table 4, second block). Own feeding rates showed no significant pattern with raw color change irrespective of sex (Table 4, third block), but after correcting change for original value, a sex-dependent relationship with the manipulation-responsive trait emerged (interaction of sex and responsive trait change in Table 4, fourth block). Corrected change in the trait that had responded to manipulation was significantly negatively related to feeding rate in males (F_1,34_ = 15.57, p < 0.001) but there was no significant relationship in females (F_1,43_ = 0.31, p = 0.583).

**Table 4:** Effects of sex, color change and their interactions on partner and own feeding rates at 10d of nestling age. Results are given both without (raw) and with (residual) correction of color change for original color value. *, p < 0.05; **, p < 0.01; SCRT, standardized change in the manipulation-responsive trait; SCNRT, standardized change in the not manipulation-responsive trait.

	Raw, partner		Residual, partner		Raw, own		Residual, own	
	F	df	F	df	F	df	F	df
Sex	0.01	1, 78	0.01	1, 79	1.68	1, 79	1.89	1, 77
SCRT	5.90*	1, 79	1.22	1, 79	3.05	1, 79	4.27*	1, 77
SCNRT	0.57	1, 78	0.00	1, 79	1.00	1, 79	1.98	1, 76
Sex $\times$ SCRT	0.09	1, 77	0.90	1, 77	2.56	1, 77	8.29**	1, 77
Sex $\times$ SCNRT	2.35	1, 76	1.04	1, 77	0.02	1, 77	0.07	1, 75

## Discussion

Our manipulations successfully changed reproductive effort and output, as shown by significant differences in offspring biomass production and the feeding rates and body mass changes in both parents in the brood size reduction group compared to the control and enlarged groups. Similar comparisons indicated that some white plumage color traits responded to the manipulation in the expected direction (less degradation in reduced than in other groups, manifested as higher brightness and UV chroma). Therefore, our data provide experimental evidence that white color degradation may reflect reproductive effort during nestling rearing.

Although both sexes reacted to the manipulation in both feeding and color deterioration, different aspects of color were affected in the two sexes, allowing us to analyze partner feeding rate in relation to the affected trait of the given sex as a possible indicator, although not a proof, of a receiver reaction. This analysis would not have been possible if the same trait had been affected by the manipulation in males and females. Moreover, the lack of correlation between male and female feeding rates within the manipulation groups indicates that negotiation between partners over care also does not confound our results. The results show that the feeding rate of the partner was significantly related to change of the color trait affected by the experimental manipulation of brood size, but not when this change was corrected for original color value. This is precisely what we would expect if the partner directly reacted to degradation in a compensatory manner. The observed degradation strongly depends on original value due to the dependence of abrasion proneness on original macrostructural intactness^28^ and also due to random measurement (observation) error. Nevertheless, the partner evidently cannot “correct” degradation for original color and it can only adjust its behavior to raw degradation. Own feeding rates after degradation, on the other hand, were related to change in the experimentally degraded color trait only in males, and the relationship appeared only when degradation was corrected for original value. This is what we expect if the structural mechanism (or other mechanism such as dirt accumulation) causing the degradation is related to male feeding patterns in the late nestling stage. Finally, no relationship with either own or partner feeding rate appeared for the “sister” color trait that did not respond to experimentally modified reproductive effort. Our inference is obviously indirect. The only way to demonstrate a causal link between color degradation and partner feeding is to directly manipulate color and we did not do such manipulations. Most of the variation in our data is experimental so we cannot statistically rule out the possibility that the correlation of color change and partner feeding is due to independent manipulation effects on both traits, although in this case we would expect the strongest relationship between color change and own feeding, and that relationship was not even present in one sex. Moreover, the change in both color and feeding is obviously gradual and our data give only a snapshot of this process that does not even involve a control stage (because the female still incubated the nestlings at the point of manipulation). Nevertheless, the feeding patterns we present are closely consistent with the hypothesis that partners adjust their feeding rates to changes in the color trait of each sex that reflects reproductive effort, but not to a similar trait that does not reflect reproductive effort.

Returning to the color degradation data, at least two particular points are worth mentioning. First, our previous studies of white reflectance degradation paid particular attention to the white wing patch. Both the short-term degradation^28^ and the actual expression^29^ of white wing patch reflectance were shown to correlate with feather macrostructural state. Moreover, reflectance was suggested to gain part of its quality-indicator value from macrostructural state, as macrostructural state reflected previous year reproductive effort and predicted current year reproductive success^29^. Our current study shows no manipulation effect on either the macrostructure (results not shown here) or the reflectance of the wing patch. As the wing patch is on the flight feathers, it is expected to be more resistant to wear and degrade over longer time scales than contour feathers^30^. In the context of our present results, previous findings indicate that the information content of the wing patch concerning current reproductive potential^29^ is due to long-term aspects of body condition (and possibly individual quality), and not short-term condition fluctuations. This is an interesting addition to the signal value of an ornament in which patch size is also very informative and functionally important in both sexes (reviewed in ref^31^).

The second interesting point concerning our results is that the only significantly affected area was the breast (UV chroma in males and brightness in females). We previously showed in females that, compared to other plumage areas, breast brightness and UV chroma have very low within-individual repeatability and great time-proportional change in expression between incubation and late nestling rearing (but particularly during incubation). We suggested that this might be because of the extreme exposure of this area to dirt and abrasion during parental duties^18^. Moreover, UV chroma has been implicated in greater area-specific deterioration than brightness in our population, with consequences for mating patterns^18,25^. Therefore, deterioration of the breast UV chroma of males by manipulated reproductive effort is not surprising. Female breast UV chroma also shows a non-significant tendency in the same direction (p = 0.133).

On the other hand, female breast brightness significantly responded to the manipulation while the homologous male trait did not (p = 0.685). Female breast feathers are much less reflective in the late nestling period than male feathers^24^, which could be because feather macrostructure differs between the sexes, with male feathers also being more resistant to wear, but note that the female breast also contains some melanin and this confounds both reflectance and degradation. A less complicated explanation is that females incubate the offspring up to 6 days of age, thereby exposing themselves to far greater amounts of dirt and wear than males. Therefore, if brightness is a less degradation-prone trait than UV chroma^32,33^, females are the sex in which we predominately expect such degradation. Finally, it is interesting that breast reflectance traits showed the smallest overall decline among the white plumage areas during nestling rearing, yet the effects of reproductive effort were the most visible on the breast. This may be because birds can easily reach their breast plumage for cleaning and ordering, but at the same time the parental care duties may leave the most visible traces on the breast.

Despite abundant correlative evidence for short-term degradation of plumage color^16–18,32,34^, little attention has been paid to the possible role of this trait type as a dynamic signal, although this is important for multiple reasons. First, manipulations of reproductive effort to induce changes in coloration at the next molt are a standard though rarely used test of condition dependence. Of the three such studies we are aware of, two measured the current-year color (or patch size) of the birds after nestling rearing and therefore after any related abrasion^35,36^, whereas one study used a generalized across-season patch size measure^37^. Any manipulation aimed to test condition-dependence of color by molt may inadvertently alter current color by the time of measurement due to degradation and therefore confound the baseline to which future ornamentation is compared. This could blur or distort the effects of the manipulation on apparent color change, so measurements of current color need to be very carefully timed at future applications of this classical method, especially if abrasion-prone traits are involved.

Second, coloration of breeding birds is most often measured when much of the investment in the breeding bout is already over (second half of nestling rearing), so color degradation may influence our assessment of the mating and success consequences and therefore fitness relevance of color^38,39^. Such an effect can indeed be detected in the UV coloration of our population^18,25^. Therefore, it would be important to widely quantify the degree of plumage color degradation during nestling rearing, and its effect on mating patterns and success correlations.

The third consequence of the effort-dependent rapid degradation we suggest here is the substantial complication of causal relations between coloration and success. Reproductive output may be determined by the dynamic balance of parental investment by both partners where the investment may depend on both own and partner ornamentation^27,40–42^. Partners have been suggested to react to changes in the provisioning of their mate^43,44^. Analogously, the assessment of within-individual color changes by the partner may transform the problem of ornament-dependent parental care to a dynamic feedback process. The relationship between ornamentation and effort is confounded by wear and it therefore changes during the reproductive bout. As a consequence of changing ornamentation and investment, partner investment and its relation to ornamentation may also change, possibly also changing partner ornamentation. Dynamic skin color traits in birds have been shown to elicit immediate responses from the receiver^45,46^, but ornamental skin colors are relatively rare. However, if within-season plumage color degradation is widespread, then the utility and interpretation of the “good parent”^41^ and differential/compensatory investment^47^ hypotheses of sexual selection depends on testing, in as many species as possible, whether reproductive investment changes with rapid shifts in partner ornamentation. Such responses in reproductive investment have been shown e.g. in both male and female blue tits *Cyanistes caeruleus*^48,49^.

In conclusion, three conditions need to be met for plumage color to qualify as a dynamic, continuously assessed signal. First, it needs to change in the relevant period (e.g. a given phase of breeding), which has been widely demonstrated. Second, its changes need to be considered by the partner, for which there is also evidence already. Finally, it needs to convey relevant information to the receiver, which our present study experimentally demonstrated. We hope that these results contribute to the refocusing of attention from static to dynamic aspects of plumage color, and to the improvement of empirical approaches and therefore better understanding of the functioning and evolution of this sexual trait type.

## Methods

### Ethics statement

We conducted this study with ethical approval by the institutional animal welfare committee of Eötvös Loránd University (permit numbers T-012/2015, T-020/2017) and with research permit from the regional nature conservation authority (permit number PE-06/KTF/920-7/2018). We conform to relevant national guidelines and regulations. The reporting of our work conforms to the ARRIVE guidelines.

### Field methods

We conducted the experiment in the breeding seasons of 2021 and 2022. We conducted a brood size manipulation in pairs of synchronously hatched broods with the same clutch size (6 or 7) and a maximum brood size difference of one. We used a similar but non-manipulated third brood as control^26,27^. We did the manipulations within five days in both years, so date variation was minimal. At the nestling age of 2 days, we transferred two nestlings from one brood to the other, and four nestlings in the reverse direction, thereby enlarging the first brood by two nestlings and reducing the second by two nestlings while keeping the ratio of own and foreign young the same in the two broods. We measured nestling masses at 2, 8 and 12 days of nestling age. At two and ten days of nestling age, we captured the parents, identified or ringed them, measured body mass (spring balance) and morphology (ruler, caliper) and conducted plumage spectrometry. We measured the reflectance of the focal plumage areas (here we analyze the male forehead patch, male and female wing patch, male and female breast) with an USB2000 spectrometer, DH2000 light source and R400-7 bifurcated fiber optic probe (Ocean Optics Europe). We recorded reflectance relative to a regularly re-measured WS-1-SS white reflectance standard and a dark standard (excluding incoming light to the detector) using the OOIBase 32 software (Ocean Optics Europe). We calculated brightness as mean reflectance from 320 to 700 nm, and UV chroma as mean reflectance from 320 to 400 nm divided by brightness^50^. Each patch was measured twice with subsequent averaging^24^. We did not correct the reflectance measures for parameters of the avian visual system primarily because these reflectance measures are apparently not processed individually but as parts of a larger ornamentation complex^24,25^.

At 10 days of nestling age, we positioned a digital camera (Panasonic HC-V100EP-K Full HD) 0.5–1 m above ground on a tripod, at least 10 m from the nest-box, to film the parental behavior of females and males. The recordings were taken between 0730 and 1200. We avoided taking recordings during rain. The presence of the camera did not disrupt feeding activity since we did not notice any lingering symptoms of disturbance. Each video was planned to be a few (10–15) minutes longer than one hour in length because robust correlations between successive hours showed that one hour recordings are representative samples of parental care in this population^27^. Three records were slightly shorter than one hour due to battery failure, the shortest being 43 min. When processing the video recordings, we calculated the feeding rate of each sex (i.e. hourly number of visits with full or partial entry through the nest-hole).

We did 8 and 10 manipulations in 2021 and 2022 respectively (involving 24 versus 30 broods together with controls). Three broods in 2021 and two broods in 2022 died early (before 9d of nestling age) and were accordingly omitted. Furthermore, we were not able to capture all parents in both early and late nestling stages, and one male was a subadult (radically different plumage) which further reduced the sample size. Sample sizes for full data were 11, 14 and 13 broods (reduced, control, enlarged) for males and 17, 18 and 13 broods for females. Finally, feeding data were not used for broods that were filmed but did not fledge any nestling because these records showed either abnormally reduced or zero feeding by one of the parents or reduced feeding by both parents (one brood in 2021 and three in 2022). The cause of these brood failures was likely external disturbance not linked to our experiment.

### Statistical analyses

Here we focus on spectral data from white plumage areas (forehead in males, wing patch and breast in both sexes). No significant correlation was found in this data set between either the brightness or the UV chroma values of white areas in either sex, irrespective of nestling age (abs(r) < 0.225), so analyzing each spectral variable separately does not represent pseudoreplication. Body mass data of the two parents were similarly uncorrelated either before or after the experiment (abs(r) < 0.167), so we considered these as independent data. Finally, number of feeding bouts was uncorrelated between the two sexes within the manipulation groups (general linear model, F_1,62_ = 0.05, p = 0.829), irrespective of year or manipulation (interactions p > 0.078).

We ran our statistical analyses in Statistica 5.5 (StatSoft, Inc) using two-tailed p values. We compared initial and final value of the given reflectance trait for each sex separately, using paired t tests. We then conducted general linear models using stepwise backward removal with reintroduction of the non-significant terms (p > 0.05) one by one (but interactions together with their constituent variables). We analyzed the initial body masses of parents with year, sex and manipulation as factors, including all interactions. We analyzed body mass changes (final minus initial) in a similar model structure but adding sex-standardized initial mass (mean of zero, standard deviation of one for both sexes) as a covariate. We analyzed total 12d nestling mass (nestling biomass production) with year and manipulation as factors, including their interaction. Because of the qualitative sexual dichromatism in nearly all spectral attributes^24^, we examined the reflectance variables for each sex separately. Initial reflectance traits were assessed with year and manipulation as factors, including their interaction. Finally, reflectance changes (final minus initial) were analyzed with initial reflectance as a covariate and year, manipulation and their interaction as discrete parameters. Post-hoc comparisons for manipulated group pairs were done by Scheffé tests in the absence of a covariate, and by re-running the interaction restricted to the given group pair in the presence of a covariate.

Finally, we analyzed post-degradation feeding rates with general linear models, pooling data from the two sexes. The original dependent variable was the feeding rate of the partner. Sex of the bearer of the focal ornament was included as a fixed factor. Continuous independent variables were the standardized (within sex, zero mean, unit variance) changes of the color variable shown to degrade in the given sex (breast UV chroma for males, breast brightness for females) and also its non-degrading “sister” trait as a control (breast brightness for males, breast UV chroma for females). Two-way interactions between sex and color change were included. We reran this model after correcting color change for original color expression (again standardized for the given sex) in a linear regression (residual change variables). Finally, we ran the same two models (raw and residual changes) by using own instead of partner feeding rate as a dependent variable.

### Supplementary Information

Supplementary Information 1.

Supplementary Information 2.

## Data Availability

The data set is uploaded as Supplementary Data S1.
