# Supplementary material for: Plumage color degradation indicates reproductive effort: an experiment
Source: Sci Rep. 2023 Oct 31;13:18770. doi: 10.1038/s41598-023-45348-0 (PMC10618437; doi:10.1038/s41598-023-45348-0)
Supplement: Supplementary file 2 — Supplementary Information 2. [file 41598_2023_45348_MOESM2_ESM.doc]

Supporting Information to

Plumage color can function as a dynamic signal

Gergely Hegyi, Miklós Laczi, Gyula Szabó, Fanni Sarkadi, János Török

**This material includes:**

Supplementary Table S1

Legend for Supplementary Data S1

**Other supplementary materials for this manuscript include**:

Supplementary Data S1

Supplementary Table S1. Original color variables and their changes in relation to year, binary manipulation (reduced versus other) and their interaction. Color change is corrected for original color by including it as a covariate. *, p<0.05; **, p<0.01; ***, p<0.001; ^a^, there is no significant manipulation effect in either year


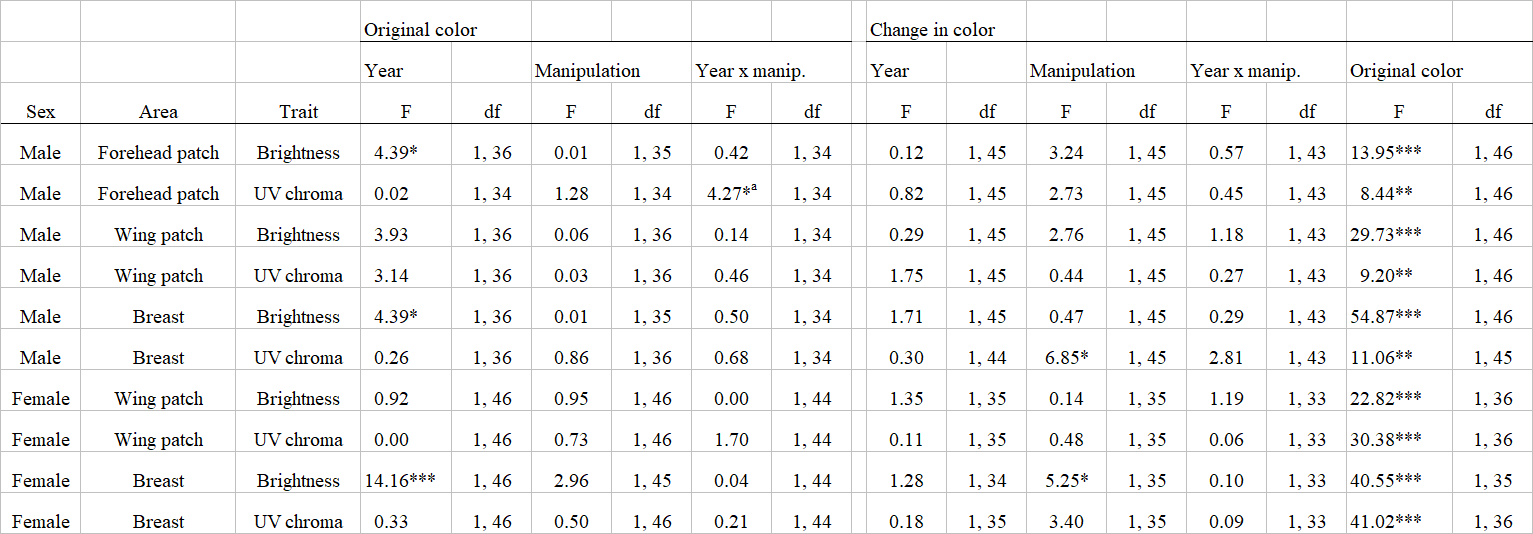


Supplementary Data S1 (separate file)

Data on brood identity, manipulations, success, offspring and parental body masses, and parental feeding rates we analyzed from our experiment done in 2021-2022. Manip, manipulation (1 reduced, 2 control, 3 enlarged); Biomass, total fledgling mass at 12d of age (unit: 0.1g); Ffeeds and Mfeeds, female and male feeding rates per hour. Parental spectrometric data follow a standard format including sex (M, male; F, female), nestling age (2d or 10d), plumage area (w, wing patch; b, breast; f, male forehead patch) and spectral variable (br, brightness; uv, UV chroma). Parental body mass data (unit: 0.1g) follow a similar format including sex (M, male; F, female) and nestling age (2d or 10d)
